# Using ChatGPT as a tool for training nonprogrammers to generate genomic sequence analysis code

**DOI:** 10.1002/bmb.21899

**Published:** 2025-05-05

**Authors:** Haley A. Delcher, Enas S. Alsatari, Adeyeye I. Haastrup, Sayema Naaz, Lydia A. Hayes‐Guastella, Autumn M. McDaniel, Olivia G. Clark, Devin M. Katerski, Francois O. Prinsloo, Olivia R. Roberts, Meredith A. Shaddix, Bridgette N. Sullivan, Isabella M. Swan, Emily M. Hartsell, Jeffrey D. DeMeis, Sunita S. Paudel, Glen M. Borchert

**Affiliations:** ^1^ Department of Pharmacology University of South Alabama Mobile Alabama USA; ^2^ Stokes School of Marine and Environmental Sciences University of South Alabama Mobile Alabama USA; ^3^ Department of Biomedical Sciences University of South Alabama Mobile Alabama USA; ^4^ Department of Biology University of South Alabama Mobile Alabama USA

**Keywords:** active learning, computational biology, computers in research and teaching, curriculum design development and implementation, genomics proteomics bioinformatics, integration of research into undergraduate teaching, original models for teaching and learning

## Abstract

Today, due to the size of many genomes and the increasingly large sizes of sequencing files, independently analyzing sequencing data is largely impossible for a biologist with little to no programming expertise. As such, biologists are typically faced with the dilemma of either having to spend a significant amount of time and effort to learn how to program themselves or having to identify (and rely on) an available computer scientist to analyze large sequence data sets. That said, the advent of AI‐powered programs like ChatGPT may offer a means of circumventing the disconnect between biologists and their analysis of genomic data critically important to their field. The work detailed herein demonstrates how implementing ChatGPT into an existing Course‐based Undergraduate Research Experience curriculum can provide a means for equipping biology students with no programming expertise the power to generate their own programs and allow those students to carry out a publishable, comprehensive analysis of real‐world Next Generation Sequencing (NGS) datasets. Relying solely on the students' biology background as a prompt for directing ChatGPT to generate Python codes, we found students could readily generate programs able to deal with and analyze NGS datasets greater than 10 gigabytes. In summary, we believe that integrating ChatGPT into education can help bridge a critical gap between biology and computer science and may prove similarly beneficial in other disciplines. Additionally, ChatGPT can provide biological researchers with powerful new tools capable of mediating NGS dataset analysis to help accelerate major new advances in the field.

## 1 INTRODUCTION

In the past decade, we have witnessed an explosive advancement in genomic sequencing technology, leading to an extensive reservoir of largely untapped sequencing data. However, that valuable information has remained, in large part, inaccessible due to biologists' general lack of programming expertise. While sequence analysis tools like The Sequence Manipulation Suite^1^ and BLAST^2^ exist and have proven to be extremely useful, the unmitigated size of datasets from whole genome sequencing has made using these applications largely impossible for non‐programmers. Until recently, biologists have been limited by either their programming capabilities or the availability of a computer scientist to assist them, which results in an unnecessary disconnect between biology and data. Therefore, the ability of a biologist to learn and teach programming languages persists as a critical roadblock with respect to unlocking unique insights embedded in large sets of sequencing data.

The importance of traditional biologists being trained in a number of computational biology skills has been highlighted in several articles published by ourselves^3^ and others.^4^, ^5^, ^6^ That said, instructors teaching computational biology usually focus their efforts on training students to utilize specialized existing programs and entirely avoid instruction aimed at teaching students how to code for themselves (and ultimately preventing them from developing new tools themselves).^7^, ^8^ The emergence of artificial intelligence (AI) resources such as ChatGPT,^9^ however, is beginning to circumvent the disconnect between biologists and the analysis of large genomic datasets by enabling trained biologists to employ skill sets that have been, until recently, almost exclusively constrained to the field of computer science.

ChatGPT is a model created by OpenAI that provides a detailed response to a written prompt of instructions given by the user.^10^ Users are able to converse back and forth with a human‐like dialogue to understand difficult topics, compose written works such as poems and essays, or various other tasks.^11^ Importantly, in addition to this, ChatGPT can also apply text interpreters to write Python codes aimed at carrying out tasks entered as dialogue by the user. This capability renders ChatGPT a strong tool for Python programming, particularly in tasks related to data handling.^12^ In short, ChatGPT can allow a user to successfully generate a unique, customized executable code (or program) by simply describing to ChatGPT what tasks they want a Python code to execute. ChatGPT's ability to write a script for someone with no prior experience with programming is quickly being recognized as a potentially invaluable educational tool, particularly in STEM.^13^

In this paper, we highlight the utility of ChatGPT in equipping undergraduate and graduate‐level students, possessing little to no prior programming experience, with the ability to write and execute fairly complex Python programs. More specifically, our group of students employed ChatGPT to generate Python scripts designed to enable rapid analysis of large RNA sequencing datasets (>10GBs). The methodology described in this work demonstrates the practicality and value of integrating ChatGPT into CURE coursework.

## 2 COURSE DESCRIPTION

Computational Genetics (IDL 590/BLY 445) is a course offered at the University of South Alabama for both graduate and undergraduate students. Similar to the course described in Smith and Harris et al. 2014,^3^ the course described herein aims to equip students in Biology and related fields with a bioinformatic foundation suitable to mine and analyze untapped information hidden in genetic sequencing data. The course consisted of 14 students: 6 graduate students, 7 undergraduate students, and 1 medical student who chose to audit the course. The undergraduates enrolled in the course had no prior experience in bioinformatics and/or coding for sequence analysis. Four of the graduate students had some previous knowledge and background in using sequence analysis tools; however, none of them had experience with writing Python programs. The semester‐long course is divided into five different sections, each offering a unique learning experience (Table 1).

**TABLE 1:** Summary of course activities and grading.

Section	Weeks	Activities description
1	1–3	Students attended lectures on genomics and related terminologies, followed by a written examination (100 points).
2	4–8	Instructor demonstrated basic online and Microsoft office‐based tools and strategies for genomic analysis. Students replicated the analyses performed by the instructor each day and submitted these as graded daily assignments (100 points).
3	6–11	Instructor assigned two papers relevant to the term research project. Students wrote a 1–2 page literature critique summarizing each paper submitting: a draft of the first critique at the end of week 6, a second draft of the 1st critique at the end of week 7, and a final critique of the 1st paper at the end of week 8 then critiqued the second paper weeks 9–11 (150 points).
4	9–12	Instructor demonstrated using ChatGPT to generate Python scripts for genomic analysis and interpretation of sequencing data. Initially daily assignments involved setting up Python and ChatGPT after which student daily assignments primarily involved generating a series of python codes aimed at carrying out specific tasks selected by the instructor (100 points).
5	13–16	Instructor coordinated a collaborative research analysis (data conceived by the instructor in advance) performed by students enrolled in the course. Students were required to maintain research notebooks documenting their work (50 points). Students were put into groups and assigned tasks to generate results and figures summarizing their findings (50 points). Instructor revised the manuscript figures with the students. Students prepared PowerPoint slides to present their final product(s) (50 points). The primary distinction between graduate and undergraduate course expectation involved graduate students being additionally responsible for compiling all the results and drafting the summary manuscript(s) for publication.

*Note*: Additional grades included student attendance (50 points) and a composite peer evaluation with students evaluating each classmate's performance at the end of the course (50 points).

### 2.1 Section 1: Genomic essentials

The first 3 weeks of the course consisted of a series of basic genetic lectures designed to ensure that all students had reviewed the concepts and terminologies necessary to understand the upcoming class research project. Topics of the lectures included DNA and RNA structure, genome composition, transcription, the functional relevance of non‐coding RNA in post‐transcriptional gene regulation, and advancements in sequencing technologies, including NGS‐based sequencing techniques.^14^, ^15^ At the end of this four‐week period, students were given an exam (100 total points) to assess their understanding of these topics, enabling the instructor to identify concepts requiring additional explanation.

### 2.2 Section 2: The basics of analyzing genomic data and daily assignments

Following the 3‐week lecture‐based review, students were assigned graded (100 total points) daily activities (to be completed and submitted before the next class) that primarily consisted of simply recreating what the instructor had demonstrated in class. Class session topics included: (1) instructions for formatting and manipulating genomic sequences using standard applications like Notepad, Microsoft Word, and Microsoft Excel, (2) demonstrating how to access and navigate various publicly available databases routinely used in analyses of genomic datasets such as Ensembl,^16^ NCBI,^17^ PubMed,^18^ and miRbase,^19^ and (3) how to perform and interpret basic sequence alignments using online and local^20^ BLAST tools.

### 2.3 Section 3: Literature critiques

In addition, students were tasked with formally critiquing two research articles directly related to the course term project that used analysis methods taught in the class. Students were given 3 weeks for each article, with the literature critique section overlapping the end of Section 2 Daily Assignments and the beginning of Section 4 ChatGPT analysis. After an initial read of the article, students would draft a summary of the paper. Following two instructor‐guided revisions, students would submit their final literature critique, with both revisions and the final submission being awarded 25 points each. Through reviewing these articles, students become better familiarized with the term project subject material, discipline‐specific writing styles and standards, and what is expected of students for the upcoming term project write‐ups and summary class manuscript.

### 2.4 Section 4: Usage of ChatGPT generated python code for genomic analysis

**FIGURE 1:**
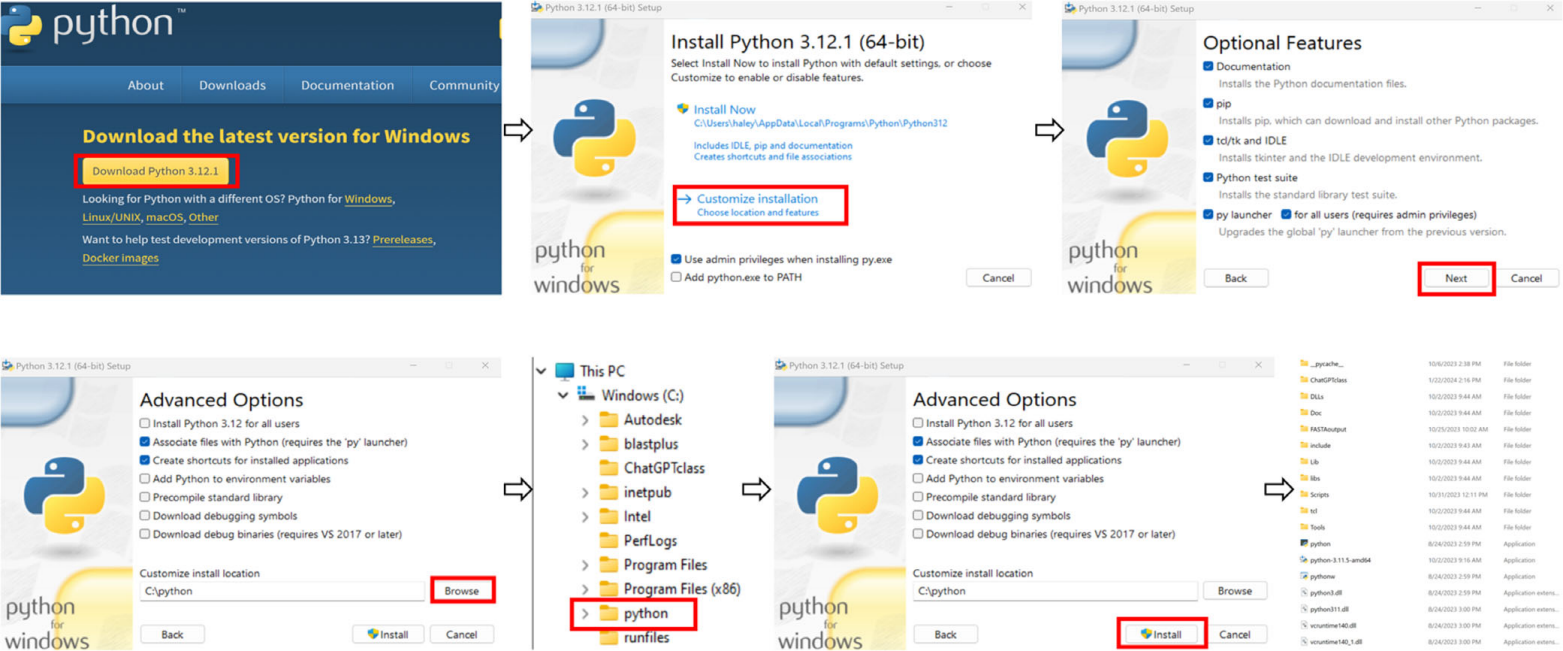
Installing Python in Windows. The steps required to install Python in Windows. Begin by downloading the latest version of Python (3.11.5). Once downloaded, install Python in the desired folder keeping the default options selected. For the purposes of this class, we created a Python folder on our C drive (C:\python), and the contents of this folder are shown in the bottom right panel. Links to proceed to each subsequent step of the installation are indicated by a red outline. Of note, python programs generated like those in this manuscript are compatible with all other operating systems. The Python website provides detailed instructions on how to download it for the user's platform of choice (e.g., MacOS).

**FIGURE 2:**
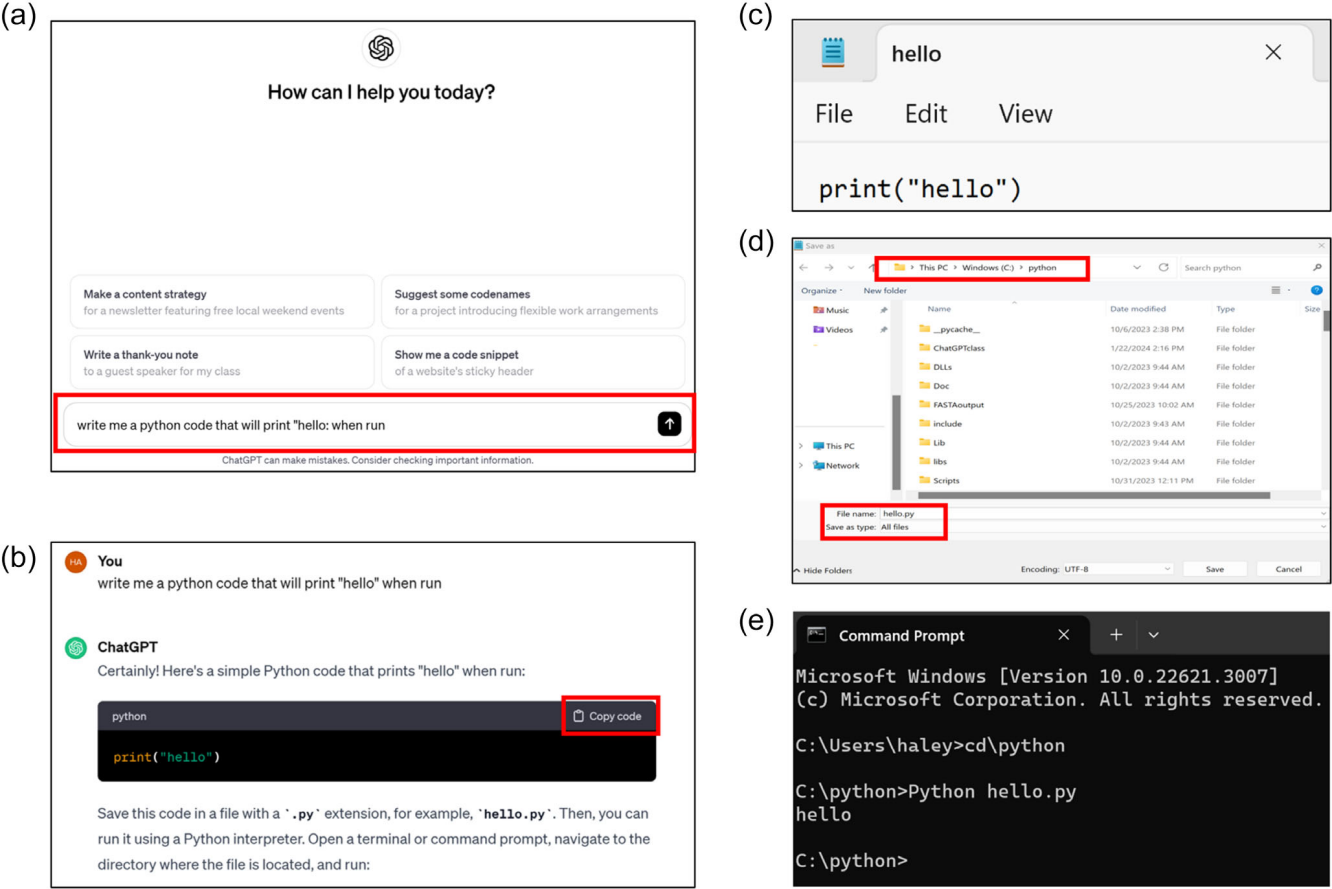
Running a ChatGPT‐generated Python script in the command prompt in Windows. (a) The ChatGPT chat window (chat.openai.com). After creating an account, the prompt for ChatGPT can be typed in the chat box shown to generate a Python code to carry out a desired function. (b) ChatGPT output response. ChatGPT will respond with a code that can be copied along with additional instructions on how to run that specific code. Selecting the “Copy code” link highlighted in red will place the code in your clipboard. (c) Python script. Open “Notepad” and paste the Python script into a new file. (d) Save the Notepad file as type “All files” and include “.py” as the extension to the end of the name of the code. The Python script shown in this example is saved as “hello.py.” Ensure that you are saving your Python script into the same folder that the Python application was installed. (e) Running the code in the command prompt. First open the Windows start button typically found at the bottom left of a Windows screen then search for “command” and open “command prompt.” Next, navigate to the folder where the Python application is installed, and the desired code has been saved (in this example this was achieved by typing “cd\python” followed by enter). After successfully navigating to the desired prompt (e.g., C:\python>) the user runs a Python script by initiating Python by typing “Python” then telling it what program to run by typing a space followed by the program name (in this example this was achieved by typing “Python hello.py” followed by enter). (See Table 2 for code details and download).

**FIGURE 3:**
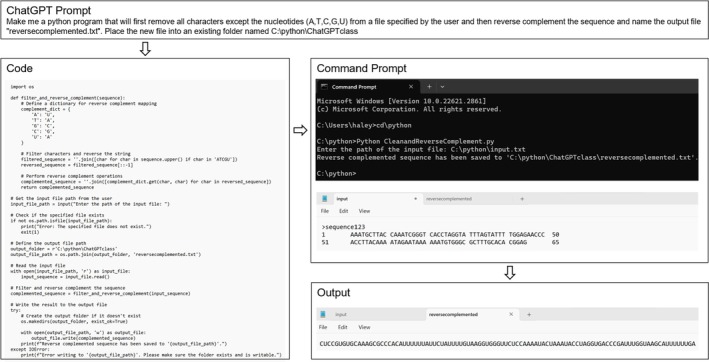
ChatGPT‐generated Python script to clean and convert a DNA sequence into its reverse complement. The prompt describes the goal of the script that was entered into ChatGPT. The code was then copied from ChatGPT, pasted into Notepad, and saved as “CleanandReverseComplement.py” in the same folder as the Python program scripts. The code was run in the command prompt, and after prompting the user to enter the input file, Python generated the output file and saved it as a text file named “reversecomplemented” in the indicated folder. (See Table 2 for code details and download).

**FIGURE 4:**
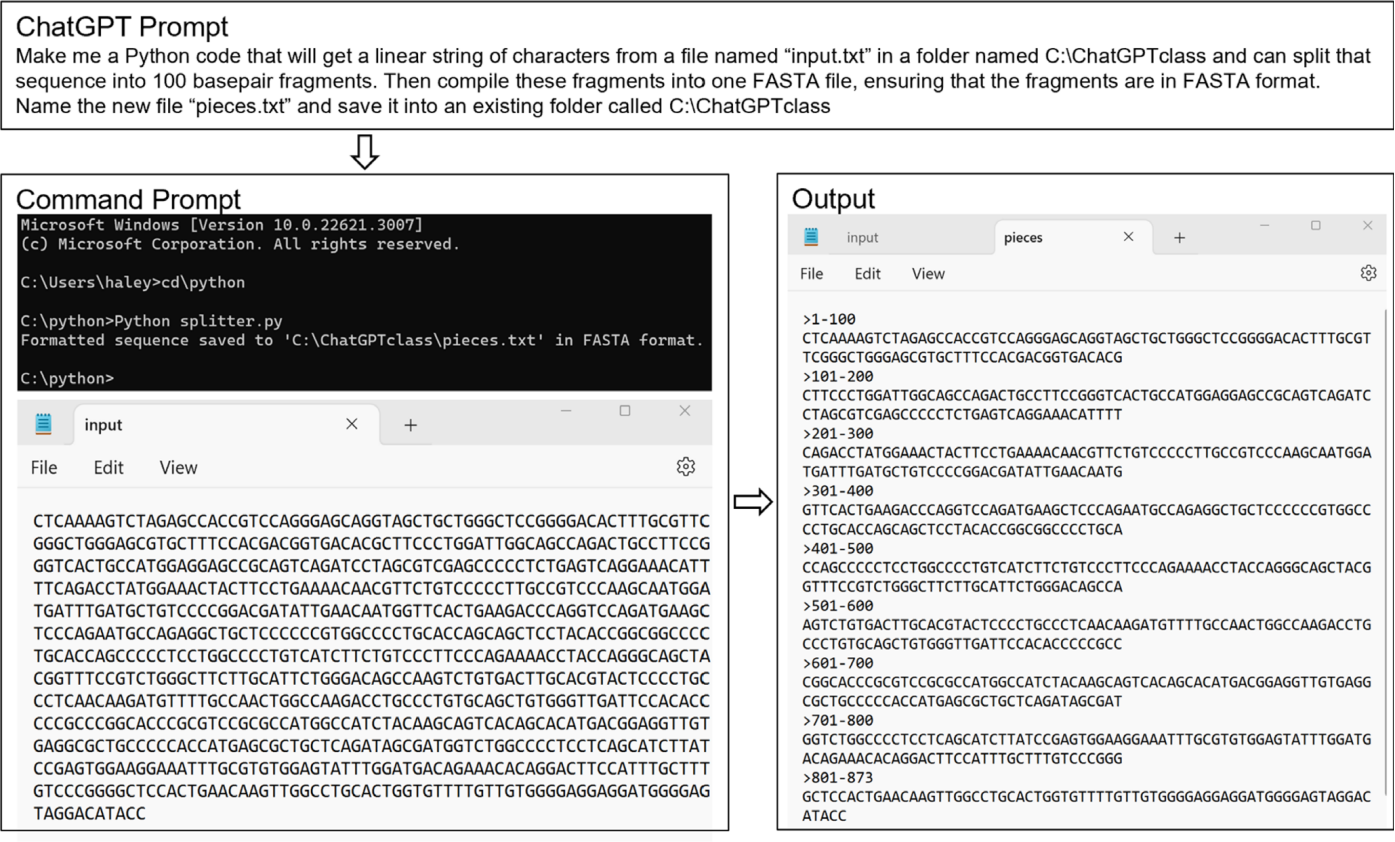
Splitting a long DNA sequence into 100 bp fragments. The prompt describes the goal of the script that was entered into ChatGPT. The Command Prompt shows the process of executing the Python code once it is saved into the correct folder. The input file was saved into the folder and named “input.txt” so that the code could identify the correct file. The output file was named “pieces.txt” and contains 100 base pair fragments in FASTA format with headers listing the positions of each fragment within the original sequence from the input file. (See Table 2 for code details and download).

**FIGURE 5:**
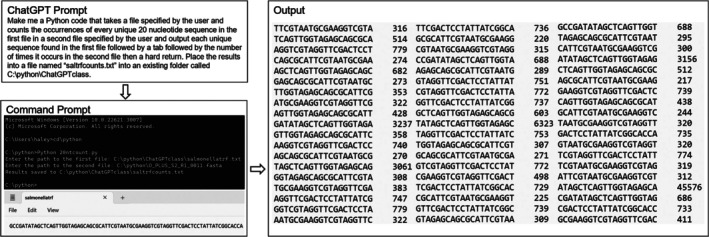
Counting the occurrence of each unique 20 nt sequence in a tRNA in a large (>1 million reads) sequencing dataset. The prompt describes the goal of the script that was entered into ChatGPT. The Command Prompt shows the code being executed along with the input tRNA sequence (from which each unique 20 nt sequence was obtained). The output lists each unique 20 nt sequence present in the Salmonella tRNA followed by the number of times that sequence occurred in the large (>1 million reads) sequencing dataset FASTA file provided (named O_PLUS_S2_R1_0011.fasta in this example). (See Table 2 for code details and download).

Following the completion of Section 2 Daily Assignments, the instructor curated and presented daily tasks (100 total points) involving the generation and execution of Python codes (Table 2). After an introduction on how to install Python and use ChatGPT (Figure 1), students began replicating tasks much like previous daily assignments. Students started with simple tasks (Figure 2) then moved into assignments involving genomic data analysis (Figures 3, 4, 5) utilizing downloaded data from NCBI, miRbase, Ensembl, and large publicly available sequencing datasets for practice.^14^, ^15^, ^16^ Importantly, the instructor emphasized that it is critical for students to understand the task they are trying to perform and that successfully completing a task has nothing to do with the coding capabilities of the student but instead rests on the background knowledge of the student in order to understand the output and confirm its accuracy. The instructor clearly conveyed to students that the most important step in utilizing ChatGPT‐generated Python codes to perform genomic analyses is to directly validate program outputs. As an example, in one ChatGPT daily task, students wrote a program to reverse complement a large set of DNA sequences and were required to randomly select three of the initial sequences and include direct confirmations that they had been properly reverse complemented in the final output in the completed assignment they submitted.

**TABLE 2:** Genomic sequencing data analysis tools created using ChatGPT.

Task	Goal	ChatGPT prompt	Python script identifier
DA08	Generate a Python script that prints “hello” when run.	Write me a Python code that will say “hello” when run	P1, S1a, S1b, S1c, S1d
DA09	Generate a Python script that can reverse complement a sequence. Generate a Python script that can remove all non‐nucleotides.	Make me a Python code that will first remove all characters except the nucleotides (A, T, C, G, U) from a file specified by the user and then reverse complement the sequence. Name the output file “reversecomplemented.txt.” Place the new file into an existing folder named C:\python\CHatGPTclass	P2, S2a, S2b, S2c, S2d, S2e, S2f, S2g, S2h, S2i, S2aa, S2aa, S2bb, S2cc, S2dd, S2ee, S2ff, S2gg
DA10a	Generate a Python script that can shuffle a given nucleotide sequence.	Write me a Python program that will get a linear string of characters from a file specified by the user and randomly shuffle the characters. Place the new linear string into a file named “scrambled.txt” into an existing folder named C:\ChatGPTclass	P3, S3a, S3b, S3c, S3d, S3e, S3f, S3g, S3h
DA10b	Generate a Python script that can split a sequence into 100 base pair fragments and compile a FASTA file containing these fragments.	Make me Python code that will get a nucleotide sequence from a file named “input.txt” in a folder called C:\ChatGPTclass and can split that sequence into 100 base pair fragments. Then, compile these fragments into one FASTA file, ensuring the fragments are in FASTA format. Name the new file “pieces.txt” into an existing folder called C:\ChatGPTclass	P4, S4a, S4b, S4c, S4d, S4e, S4f, S4g, S4h
T1 DA11 DA12 DA13	Generate a Python script that can look for sequence similarity of a specified nucleotide length between two sequences.	Make me a Python code that takes a file specified by the user and counts the occurrences of every unique 20 nucleotide sequence in the first file in a second file specified by the user and output each unique sequence found in the first file followed by a tab followed by the number of times it occurs in the second file then a hard return. Place the results into a file name “saltrfcounts.txt” into an existing folder called C:\python\ChatGPTclass	P5, S5a, S5b, S5c, S5d, S5e, S5f, S5g, S5h, S5i
DA14	Generate a Python script that can count the number of reads in a FASTA file.	Make me a Python code that will count the number of reads in a FASTA file specified by the user. Create a new .txt file that reports the number of reads and name the new file “filename_x_reads.txt” where “filename” is the name of the file that was input, and “x” is the number of reads reported. Save the output file to a folder named C:\python\ChatGPTclass	P6, S6a, S6b, S6c, S6d, S6e, S6f, S6g, S6h
DA15 DA16 DA17	Generate a Python script that can extract the names of reads from a BLAST output file. Generate another Python script that will extract those reads from a FASTA file then make a new FASTA file containing these reads.	Make me a Python program that can take a BLAST output file specified by the user and extract the unique values in column A. Place these names into a new .txt file called “extractedreads_#.txt” into an existing folder C:\python\ChatGPTclass where # equals the number of reads were extracted. Make me a Python code that will find each read listed in a .txt file specified by the user in a FASTA file specified by the user. Copy the sequence that follows each read and paste it into a new FASTA file. Make sure that the generated FASTA file is in FASTA format. Name the new file “readsequences.fasta” and place it into an existing folder C:\python\ChatGPTclass	P7, S7a, S7b, S7c, S7d, S7e, S7f, S7g P8, S8a, S8b, S8c, S8d, S8e, S8f, S8g, S8h
DA18a	Generate a Python script that can identify hits to the same read in two different BLAST output files then make a new output file showing reads found in both files and their associated values.	Make me a Python code that reads two files specified by the user, searches for matching values between the first column of the first file and the first column of the second file. When a match is found, it creates a new line in the output file with the matching name, followed by the values from both files between the third and fourth tab. Save the output file as “samehits.txt” into an existing folder C:\python\ChatGPTclass	P9, S9a, S9b, S9c, S9d, S9e, S9f, S9g
T2	Make a Python code that removes reads from a FASTA file.	Make me a Python code that will find each read listed in a .txt file specified by the user in a FASTA file specified by the user. Omit this read along with the sequence that follows and only copy the sequences the follows each read that is not a match and paste it into a new FASTA file. Make sure that the generated FASTA file is in FASTA format. Name the new file “uniquereadseqs.fasta” and place it into an existing folder C:\python\ChatGPTclass	P10, S10a, S10b, S10c, S10d

*Note*: Task: Name of daily assignment (DA) or task (T) that was assigned in class by the instructor. Goal: Describes what the Python script should achieve. ChatGPT Prompt: Exact verbiage used in the prompt given to ChatGPT allowing it to generate the desired code. Python Script Identifier: Arbitrary ID (as found in GitHub) given to each code generated that achieved the desired goal but was generated by inputting different student verbiage. P denotes the exact script generated using the prompt written in the table. S denotes other scripts that also achieved the desired goal but were generated by different students inputting their own unique verbiage. Scripts can be obtained from GitHub according to the Python Script Identifier. https://github.com/glen‐borchert/ChatGPTgeneratedPythonCodes‐ForBioinformaticAnalysis.

### 2.5 Section 5: Research analysis

After demonstrating proficiency with generating and executing Python codes using ChatGPT, the class focus switched to a collaborative research project coordinated by the instructor involving the analysis and interpretation of real RNA sequencing of *Salmonella* infected with P22 bacteriophage. In an attempt to identify new RNA genes, groups of four students were assigned specific tasks to mine RNA sequencing data by employing ChatGPT‐generated Python codes (Table 2). The three graduate students who scored the highest on the exam were chosen to be group leaders. One group was led by a post‐doctoral fellow from the Borchert Lab. The remaining students were randomly assigned to a group leader by the instructor. Each group assembled tables and/or figures (revised with guidance from the instructor) summarizing their findings (50 points). Students then presented their finalized figures and tables in a PowerPoint presentation to the class (50 points). Additional points were awarded as a part of the research analysis section for (1) keeping a research notebook (50 points), (2) student peer evaluations (50 points) (responses were submitted to the instructor of the course, kept confidential, and not used for research purposes), and (3) attendance (50 points). Of note, the primary distinction between graduate and undergraduate student expectations was that graduate students were responsible for managing the drafting and preparing of the final manuscript(s) for publication. Graduate students were offered the opportunity to be the first author on one of the two manuscripts resulting from our work. Of the seven graduate students, three volunteered to be the first author for this particular manuscript and worked equally together to warrant co‐first authorship. The rest of the students in the class were paired into teams individually tasked with providing a complete draft, accompanying legend, and text description of at least one of the tables or figures included in each manuscript to warrant their co‐authorships.

## 3 METHODS

### 3.1 Installing python and setting up a ChatGPT account

With assistance from the instructor, Python (version 3.11.5) was installed by each student onto either their personal computer or those provided in the classroom computer lab according to the download instructions on the Python download website^21^ (Figure 1). Each student also created their own personal ChatGPT account^9^ (*version 3.5*). To acclimate students with using ChatGPT and running Python, the instructor demonstrated how to generate a simple code that would print “hello” in the command prompt when the Python code was executed (Figure 2). In the ChatGPT chat box students typed, “make me a Python code that will print ‘hello’ when run”. ChatGPT then responded with a Python code that was copied and pasted into Notepad. Then, the code was saved as “hello.py” into the same folder that the Python application was installed. The functionality of the code was validated by running the Python script in the command prompt. Note, a Python script can only be run if the script is in the same folder as the Python software. To run the Python script the command prompt was opened then students navigated to the directory where the Python program scripts were installed and typed the following command: “Python hello.py” and pressed enter to execute the script. Step‐by‐step instructions for both installing Python and using ChatGPT to write and execute the code are detailed in Figures 1 and 2. Note, (1) it is important to provide complete and accurate instructions to ensure that generated codes accurately reflect intended functionality and (2) desired outcomes must be directly confirmed in output files.

## 4 RESULTS

### 4.1 Completing bioinformatic tasks using ChatGPT to generate Python scripts

Students used ChatGPT to craft Python codes to achieve tasks typically requiring established bioinformatic tools. For an initial, simple DNA sequence manipulation, students prompted ChatGPT to write a Python code to remove all non‐nucleotides and reverse complement a given DNA sequence (Figure 3). This sequence manipulation tool is necessary to decrease the clean‐up time for sequencing files and makes it possible to clean and reverse complement files that are too large to be accessed using programs such as Microsoft Word and The Reverse Complement Tool.^1^ All students successfully generated code achieving the desired result. While most students generated individual Python codes to (1) clean up the sequences and then (2) reverse complement the cleaned sequence, one student successfully generated a code that could perform both tasks simultaneously.

Being able to split a large sequence into smaller fragments allows for a more manageable analysis of sequencing data. Using the same process illustrated in Figure 3, students generated a Python code that divides a long string of nucleotides (nt) into 100 nt segments and compiles these segments into one FASTA file with the nucleotide positions as the header for each segment (Figure 4). FASTA format is the standard format for DNA sequences (to be utilized by bioinformatic programs) in which a greater than sign signals the start of a new sequence and is followed by a title called a header which ends with a hard return immediately followed by a linear string of nts^22^ (see Figure 4 output). The majority of students were able to successfully achieve the desired result by executing their generated code. Students unable to generate a functional code were still expected to turn in an image of their prompt, the code generated, and their result for full credit and also to obtain and run a functional code provided by another classmate.

To attempt a more complex task, students were asked to screen a large FASTA file containing millions of unique *Salmonella* RNA sequencing reads for any 20 nt perfect matches to any part of an ~70 nt *Salmonella* tRNA and to count the number of times each 20 nt piece occurred (Figure 5). Identifying short matches between sequences is biologically relevant and a common theme in many bioinformatic analyses. Only 4 students successfully attained the desired output. Again, students unable to generate a functional code were still expected to turn in an image of their prompt, the code generated, and their result for full credit and also to obtain and run a functional code provided by another classmate.

### 4.2 Analyzing large RNA sequencing files using ChatGPT‐generated python code

**FIGURE 6:**
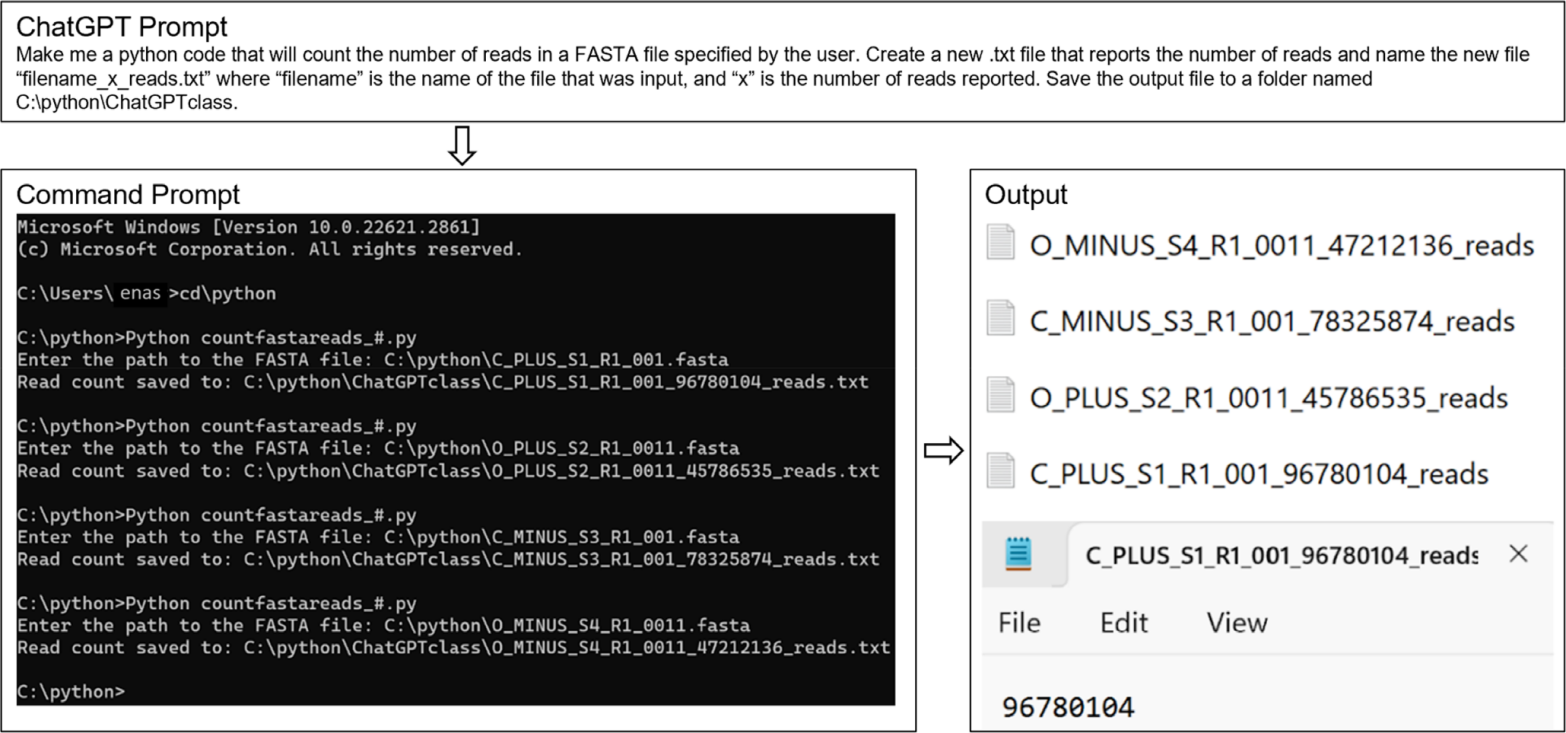
Counting the number of FASTA reads in a FASTA file. The prompt describes the goal of the script that was entered into ChatGPT. The Command Prompt shows the code being executed along with the input files input by the user. The output that was generated shows the number of reads in each FASTA by renaming the file as “input file name_# of reads.fasta.” (See Table 2 for code details and download).

Excitingly, students were able to utilize ChatGPT to analyze real RNA sequencing datasets. Large sets of FASTA files containing reads from RNA samples isolated from *Salmonella* were analyzed. Students began by prompting ChatGPT to make a code that would count the number of FASTA reads in a specified FASTA file (Figure 6). Using the generated code, the number of reads in a 10–20 gigabyte (GB) FASTA file can be reported in a matter of seconds, making it easier to calculate reads per million (RPM) (a common metric used to report the levels of RNA expression). All students were able to successfully generate code to execute this function, although three of the 12 codes proved to be markedly more efficient than the others.

**FIGURE 7:**
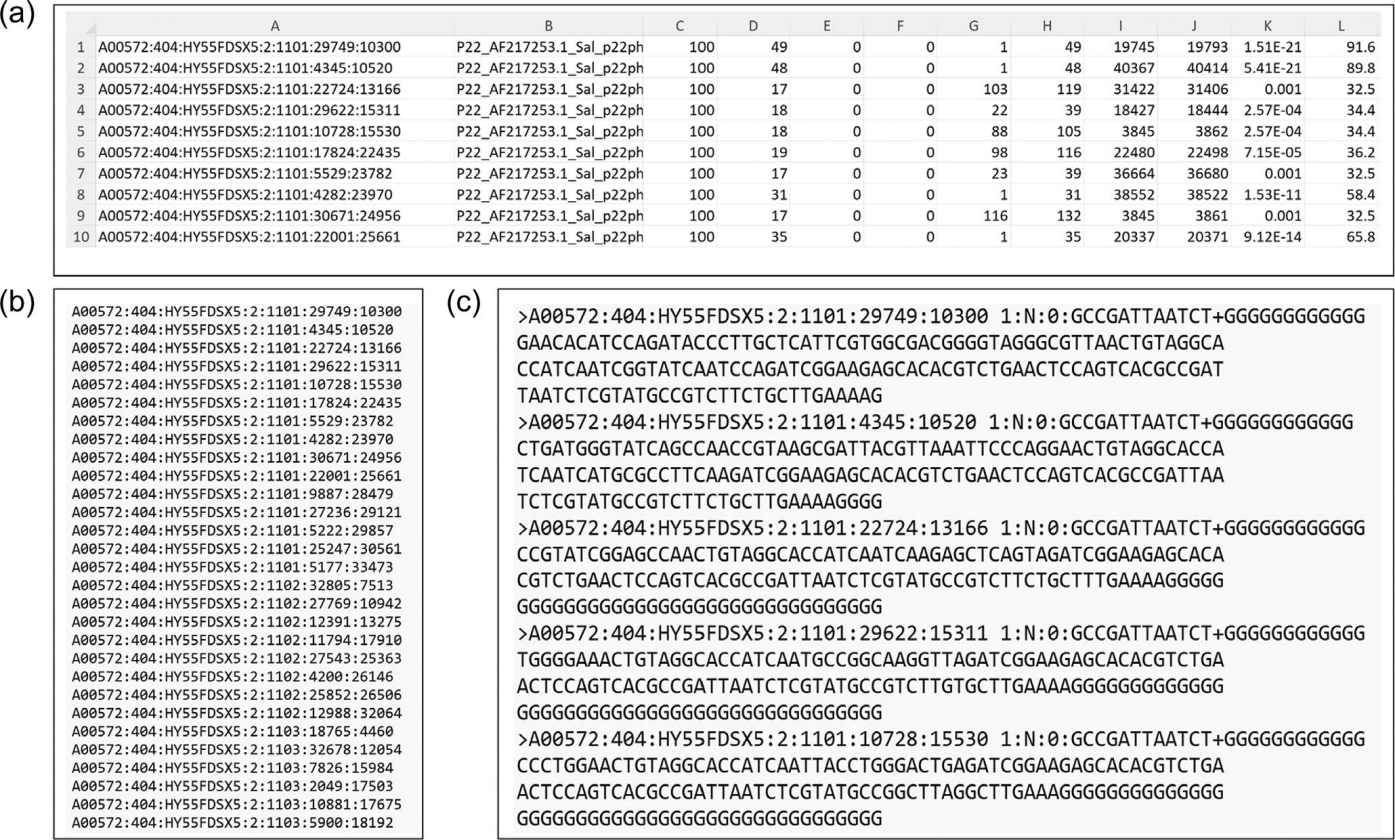
Extracting read names from a BLAST output file and its corresponding sequence from a FASTA file. An example of reads that were extracted during our data analysis for the class project. (a) Standalone BLAST results between RNA sequences isolated from *Salmonella* and the P22 genome. Column A: Name of read from *Salmonella* RNA sequencing results (Query), Column B: The sequence that our RNA reads aligned to (all of which were the P22 genome) (Subject), Column C: Percent Identity of alignment, Column D: Length of alignment, Column E: Mismatches in the alignment, Column F: Gaps in the alignment, Column G: Start position of *Salmonella* read that aligned, Column H: End position of *Salmonella* read that aligned, Column I: Start position of the P22 genome region that aligned to the *Salmonella* Read, Column J: End position of the P22 genome region that aligned to the *Salmonella* Read, Column K: E value, Column L: Alignment score. (b) List of read names extracted from Column A of the BLAST output file using a Python code generated by ChatGPT. (c) List of corresponding sequences extracted from the full FASTA file then saved in a new FASTA file just containing these sequences by a second Python code generated by ChatGPT (See Table 2 for code details and download).

The codes most directly applicable to the class research project involved analysis of Basic Local Alignment Sequence Tool (BLAST) output data. Standalone BLAST is commonly used for finding regions of similarity between two sequences.^2^ For the class project, a standalone BLAST was performed comparing FASTA files containing millions of RNA sequencing reads to the entire *Salmonella* or *Salmonella*‐infecting bacteriophage (P22) genome. Resulting BLAST alignment data is output as an excel file (.xlsx) which contains the sequence name from the FASTA file (column A), the genome name that matched to the read from the FASTA file (column B), the percent match between the two sequences (column C), the length of the sequence match (column D), the number of mismatches in the sequence match (column E), the number of gaps between the sequences (column F), the start position of the FASTA read (column G), the end position of the FASTA read (column H), the genome start position of the sequence match (column I), the genome end position of the sequence match (column J), and the expected probability (e‐value) (column K) (example of BLAST output shown in Figure 7). Output files from the class analysis included millions of sequence matches making these files and their corresponding sequences inaccessible due to their large sizes as shown in Figure 6. Therefore, students were tasked with creating codes that would extract the unique names of reads from the BLAST output file (.xlsx) then identify and extract the corresponding sequences of these reads from the original FASTA file (Figure 7). Codes capable of successfully completing these tasks were only generated by three students; however, like previously stated, students who were able to generate working codes shared these with the rest of the class, fostering a collaborative work environment among students.

## 5 DISCUSSION

In this paper, we present an original and innovative approach to teaching programming skills to biology students by utilizing ChatGPT as a bioinformatics tool for NGS analysis. It has become critical for biology and other related fields for students to learn the necessary programming skills to complete bioinformatic tasks as data sets grow larger in size and complexity. The primary objective of the course was to provide students (i) with a basic knowledge of genomics and then (ii) the fundamental tools routinely used in conducting comprehensive whole genomic and transcriptomic analysis. Excitingly, we have successfully exceeded the course expectations by not only imparting basic knowledge of genetics and its application in sequencing data analysis but also equipping students with the ability to utilize ChatGPT as a novel bioinformatics tool to generate Python code to interpret and analyze large datasets.

As a part of the CURE curriculum, students actively participated in a real‐world research project enabling them to apply the computational skills learned in class to analyze and interpret large sets of RNA sequencing data isolated from *Salmonella* (data to be published at a later date). Students were split into 4 groups and tasked with analyzing 1 of 4 RNA sequencing files. Even though students were in groups, each student was required to attempt each assignment for the data analysis independently then corroborate their results with their teammates'. Interestingly, we found that despite each student giving ChatGPT similar (and at times even the exact same) prompts, ChatGPT always produced slightly different programs and routinely only some of these were capable of completing the desired task. We observed no correlation between which student prompts typically generated functional codes and which did not, but did find that as task complexity increased so did the number of students unable to complete the assignment with the code that they had generated using ChatGPT. We believe this is primarily due to the adaptive, flexible nature of AI and will become increasingly less of an issue as AI platform performances improve over time. That said, because students were working parallel to one another attempting to achieve the same task, in instances where one team member's code worked (as confirmed by direct student validation) but the others' did not, or when one code was found to be significantly more efficient (e.g., decreased run time), codes were shared among the students to complete the assignment. Due to this, we suggest larger class sizes are actually beneficial in terms of successful completion of collaborative group research projects as we found that having multiple students attempt to generate the same code in parallel allowed the class to continue to push forward without setbacks and produce publishable results in the allotted time frame.

In summary, we believe that actively integrating AI powered programs like ChatGPT into undergraduate education has the potential to bridge a critical gap between biology and computer science and may prove similarly beneficial in other disciplines. We also suggest the ability of ChatGPT to provide current biological researchers with new tools capable of mediating NGS analysis will likely garner significantly more attention and help accelerate major new advances in this field in the near future. In addition to this, we found that having students work on the same task in parallel allowed the class to continue to push forward without setbacks and produce publishable results in the allotted time frame when one team member's code worked but the others' did not. In conclusion, although this pedagogical method has not been formally assessed, this pilot course shows that ChatGPT can be successfully utilized in an undergraduate class setting to train nonprogrammers to independently develop computational resources suitable for carrying out a real‐world research analysis.

## Data Availability

Generated Codes supporting our findings are publicly available on GitHub using the URL: https://github.com/glen‐borchert/ChatGPTgeneratedPythonCodes‐ForBioinformaticAnalysis.
